# Monocyte to High-Density Lipoprotein Ratio: A Novel Predictive Marker of Disease Severity and Prognosis in Patients With Neuromyelitis Optica Spectrum Disorders

**DOI:** 10.3389/fneur.2021.763793

**Published:** 2021-10-27

**Authors:** Jinwei Zhang, Yanfei Li, Yongyan Zhou, Kaixin Wang, Chunyang Pan, Yi Zhao, Haojie Xie, Ranran Duan, Zhe Gong, Yanjie Jia

**Affiliations:** Department of Neurology, The First Affiliated Hospital of Zhengzhou University, Zhengzhou, China

**Keywords:** neuromyelitis optica spectrum disorders, monocyte to high-density lipoprotein ratio, prognosis, severity, expanded disability status scale

## Abstract

**Background and Purpose:** To investigate the association of monocyte to high-density lipoprotein ratio (MHR) with disease severity and prognosis in patients with neuromyelitis optica spectrum disorders (NMOSD).

**Methods:** This retrospective study included 125 patients with NMOSD. Demographic and clinical parameters, including the MHR, were assessed. The initial Expanded Disability Status Scale (EDSS) score and relapse rate were used to evaluate disease severity and prognosis, respectively. Correlations between MHR and disease severity and relapse rate were analyzed. The predictive value of MHR for prognosis was evaluated using receiver operating characteristic (ROC) curve analysis.

**Results:** Compared with the low MHR group, the initial EDSS score (median 4.5 vs. 5.5%, *P* = 0.025) and relapse rate (51.61 vs. 30.16%, *P* = 0.015) were significantly higher in the high MHR group. MHR was positively correlated with the initial EDSS score (*r* = 0.306, *P* = 0.001). Multivariate analysis showed that MHR was significantly associated with severity (odds ratio = 7.90, 95% confidence interval [CI] = 1.08–57.82, *P* = 0.041), and it was a significant predictor of disease prognosis (hazard ratio = 3.12, 95% CI = 1.02–9.53, *P* = 0.046). The median relapse interval of the high MHR group was 24.40 months. When the MHR was higher than 0.565, the risk of relapse was high [sensitivity, 33.3%; specificity, 91.9%; area under the ROC curve, 0.642 (95% CI = 0.54–0.74, *P* = 0.007)].

**Conclusion:** MHR is a novel predictive marker of disease severity and prognosis in patients with NMOSD. Early monitoring and reduction of MHR may allow earlier intervention and improved prognosis.

## Introduction

Neuromyelitis optica is an autoimmune demyelinating disease of the central nervous system that is characterized by acute optic neuritis and transverse myelitis occurring simultaneously or continuously (1–3), with an estimated prevalence of 1–2 per 100,000 people. Approximately 80% of patients have specific antibodies to astrocyte aquaporin 4 (AQP4), and this is one of the key diagnostic criteria (4–6). Patients with neuromyelitis optica spectrum disorders (NMOSD) have severe immune-mediated attacks that usually lead to severe residual disability and a high relapse rate (7). Therefore, accurate prediction of relapse is important to help clinicians initiate early preventive treatment and improve patient prognosis (8, 9).

As an important effector cell of the innate immune response, monocytes play a key role in the pathogenesis of autoimmune-related central nervous system diseases, including NMOSD (10–13). Studies have shown that anti-AQP4 antibodies can stimulate astrocytes to release chemokines, recruit monocytes and promote their activation, enhance the natural immune response, and destroy the blood-brain barrier, which plays a key role in accelerating the formation of NMOSD lesions (14–17). High-density lipoprotein (HDL) is considered an anti-inflammatory factor that has immunomodulatory and antioxidant effects on endothelial cells (18–20) and can prevent the production of pro-inflammatory cytokines. Studies have shown that low HDL is related to disease activity and disability in patients with AQP4-positive NMOSD, which may be associated with the decrease in the levels of apolipoprotein (apo) A-I, the main component of HDL in serum (21, 22).

The monocyte to high-density lipoprotein ratio (MHR) is a novel marker that reflects the degree of inflammation and oxidative stress. Many studies have shown that the MHR is closely related to the occurrence, development, and prognosis of cardiovascular, cerebrovascular (23–25), immune system (26), and rheumatic diseases (27–30). For example, one study found that the MHR was significantly higher in patients with multiple sclerosis than in healthy controls and that it was related to disease severity and disability, suggesting that the MHR may be used as an independent index to predict disability (31). However, the correlation between MHR and prognosis and relapse in patients with NMOSD remains unclear. The purpose of this study was to explore the relationship between MHR and disease severity and prognosis in patients with NMOSD and to determine the best cutoff value of MHR to predict the prognosis of patients with NMOSD.

## Materials and Methods

### Patients

**Figure 1:**
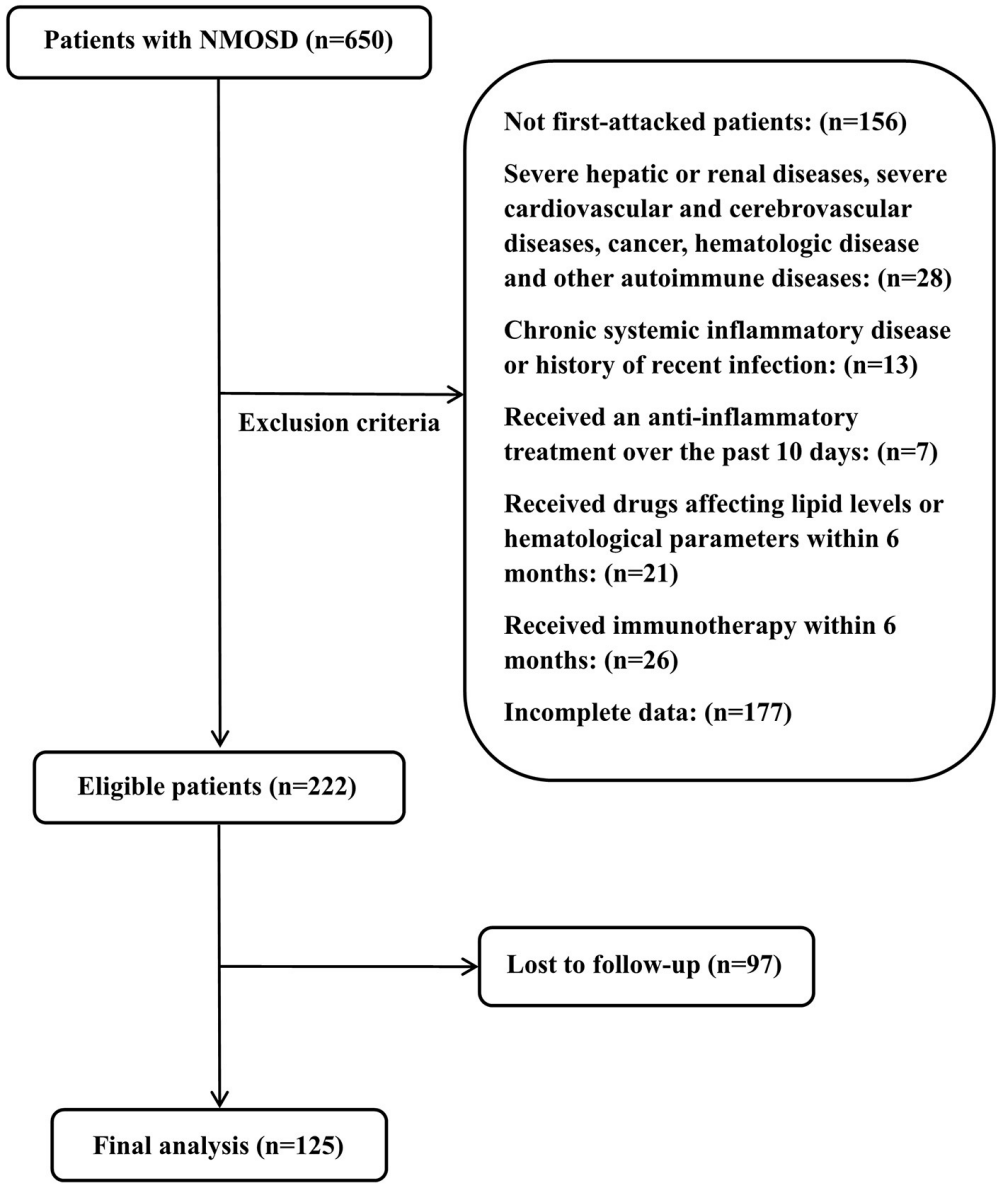
Flow chart of the study population. NMOSD, neuromyelitis optica spectrum disorders.

This study was approved by the Ethics Committee of Zhengzhou University (2019-KY-018). All patients provided written informed consent to participate in this study. In this retrospective study, we collected the clinical data of 650 patients newly diagnosed with NMOSD from September 2013 to June 2020 at the First Affiliated Hospital of Zhengzhou University. The inclusion criteria were: (1) diagnosed with NMOSD for the first time according to the 2006 Wingerchuk standard or the 2015 McDonald NMOSD international general diagnostic standard (32, 33); (2) no serious liver or kidney damage, serious cardiovascular or cerebrovascular diseases, malignant tumors, blood system diseases, or other immune diseases; (3) no chronic systemic inflammatory diseases or history of recent infection; (4) did not receive treatment with anti-inflammatory drugs within 10 days before admission, no lipid-lowering treatment within 6 months before admission, and were not taking drugs that affect the number of white blood cells and blood lipid levels; (5) did not receive immunosuppressant treatment within 6 months before admission, and (6) had complete clinical data and follow-up data. A total of 125 patients met these criteria and were included in this cohort study. The specific screening process is shown in Figure 1.

### Data Collection

Clinical data were obtained through a retrospective review of the hospital electronic case system. The following clinical information was collected: gender, age, clinical phenotype at onset, comorbidities, blood cell count, triglyceride (TG), total cholesterol (TC), low-density lipoprotein (LDL), HDL, MHR, erythrocyte sedimentation rate (ESR), C-reactive protein (CRP), anti-AQP4 antibody status, spinal magnetic resonance imaging (MRI), and treatment plan.

Blood samples were collected from the antecubital vein at 8:00 am after an overnight fast and stored on ice before testing (within 30 min after collection). Blood samples were analyzed in the biochemistry laboratory of the hospital. Blood cell count and all biochemical parameters were determined by standard methods, and the enzyme kit (Sigma-Aldrich, Saint Louis, MO, USA) was used to evaluate fasting TC, TG, LDL, and HDL levels. The anti-AQP4 antibody status in serum or cerebrospinal fluid samples was analyzed by the cell-based assay method. MRI of the spinal cord was performed using a 3T MAGNETOM Skyra scanner (Siemens Healthcare, Erlangen, Germany) in the Department of Magnetic Resonance of the hospital at admission. Longitudinally extensive transverse myelitis (LETM) was considered as extending 3 or more vertebral segments on T2-weighted imaging (33). When more than one test was performed during hospitalization, the data from the first test were included in the analysis. All tests were conducted according to the manufacturer's instructions, and the inspectors were blinded to the diagnosis or clinical symptoms.

### Clinical Assessment

To calculate the extended disability status scale (EDSS) score at the time of admission, at least two professional neurologists carefully reviewed the patient's clinical records. This was recorded as the initial EDSS score, which was used to evaluate disease severity. The baseline EDSS scores of all patients before the first attack were considered normal. Patients in the cohort were divided into those with mild disability (EDSS score 0–3.5) or moderate/severe disability (EDSS score 4–9.5) (34–36).

The main index for evaluating prognosis was the relapse rate, which was defined as new or relapsed neurological symptoms caused by demyelinating diseases of the central nervous system without fever or infection, lasting at least 24 h, and increasing the existing EDSS score of patients by 0.5 points (37). Follow-up data were obtained by a clinical visit or telephone review every 6 months.

### Statistical Analysis

Statistical analysis was performed using SPSS version 26.0 (International Business Machines Corporation, Chicago, Illinois, USA). According to the median MHR, the cohort was divided into two groups, and the classification data were expressed as percentages (%). The chi-square test or Fisher's exact test was used to compare the two groups. The Kolmogorov-Smirnov test was used for normality testing, and the measurement data conforming to normal distribution were expressed as mean and standard deviation; the independent sample *t*-test was used for comparison between the two groups. Data with non-normal distribution were expressed as median and interquartile range, and the Mann-Whitney *U* test was used for comparison between the two groups. The correlation between MHR and other clinical indices and the initial EDSS score was obtained by Spearman correlation analysis. Patients in the cohort were divided into those with mild disability (EDSS score 0–3.5) or moderate/severe disability (EDSS score 4–9.5) (34–36). We used a binary logistic regression model to analyze the correlation between MHR and other clinical-related indicators and disease severity. Variables with *P* < 0.2 in univariate analysis and those that were closely related to dependent variables in the clinic were included in the multivariate model, and the results were expressed with the odds ratio (OR) and 95% confidence interval (95% CI). The Kaplan-Meier curve was used to analyze whether different MHRs had an independent influence on the time of the first relapse, and univariate Cox survival analysis was used to screen the variables with *p* < 0.2. Then the multivariate regression model was used to analyze whether MHR was a predictor of relapse in patients with NMOSD. The results were expressed as the risk ratio with the 95% CI. We used a receiver operating characteristic (ROC) curve to analyze the predictive value of MHR for disease prognosis, determine its optimal critical value, and calculate the area under the curve to evaluate the accuracy of the cutoff value. Differences were considered statistically significant at *p* < 0.05.

## Results

### Demographic and Clinical Characteristics of Participants

A total of 125 patients with NMOSD were enrolled in this cohort study (Figure 1). The average age at onset was 42.53 ± 15.06, 106 patients were women (84.80%), 75 were positive for anti-AQP4 antibody (60%), and the average follow-up time was 37.87 (20.27–51.85) months. The most common clinical phenotype at onset in the whole cohort was optic neuritis (72%), and 66.40% of patients had extensive transverse myelitis on MRI of the spinal cord. To better evaluate the relationship between different MHRs and disease severity and prognosis, we divided patients into MHR ≤ 0.40 and MHR > 0.40 groups, according to the median MHR. There were no significant differences in age, gender, clinical phenotype and hypertension between the two groups. Compared to the low MHR group, the high MHR group had significantly lower TC levels (3.93 vs. 4.44 mmol/L, *P* = 0.005), and there were significantly more patients with LETM on MRI of the spinal cord (47 vs. 36, *P* = 0.027). Other clinical parameters, such as lymphocyte count, TG, LDL, ESR, and CRP, were not significantly different between the two groups. There was a significant difference in the initial EDSS score between the two groups (5.5 vs. 4.5, *P* = 0.025). The relapse rate of the high MHR group was higher than that of the low MHR group (51.61 vs. 30.16%, *P* = 0.015). Other demographic and clinical characteristics are shown in Table 1.

**Table 1:** Demographic and clinical parameters of patients with NMOSD.

	**Total (*n* = 125)**	**MHR ≤ 0.40 (*n* = 63)**	**MHR > 0.40 (*n* = 62)**	***P***
Age at onset, years, mean ± SD	42.53 ± 15.06	43.51 ± 14.10	41.53 ± 16.04	0.527
Gender, female, n (%)	106 (84.80)	55 (87.30)	51 (82.26)	0.432
Clinical phenotype at onset, n (%)
Optic neuritis	72 (57.60)	32 (50.79)	40 (64.52)	0.121
Acute myelitis	38 (30.40)	21 (33.33)	17 (27.42)	0.472
Optic neuritis+ acute myelitis	10 (8.00)	5 (7.94)	5 (8.06)	0.979
Other combinations	25 (20.00)	15 (23.81)	10 (16.13)	0.283
Hypertension, *n* (%)	10 (8.00)	5 (7.94)	5 (7.81)	0.979
Anti-AQP4 status, n (%)
Positive	75 (60.00)	38 (60.32)	37 (59.68)	0.942
Negative	50 (40.00)	25 (39.68)	25 (40.32)	
Laboratory test results, median (IQR)
Monocytes, × 10^9^/L	0.49 (0.34–0.63)	0.34 (0.25–0.46)	0.59 (0.52–0.79)	<0.001^*^
Lymphocytes, × 10^9^/L	1.50 (1.08–2.20)	1.38 (1.00–2.15)	1.59 (1.14–2.49)	0.133
TC, mmol/L	4.29 (3.51–5.01)	4.44 (3.90–5.23)	3.93 (3.24–4.88)	0.005^*^
TG, mmol/L	1.08 (0.72–1.59)	1.03 (0.68–1.72)	1.10 (0.76–1.50)	0.743
HDL, mmol/L	1.23 (1.01–1.51)	1.37 (1.15–1.70)	1.07 (0.90–1.34)	<0.001^*^
LDL, mmol/L	2.70 (1.96–3.32)	2.79 (2.07–3.41)	2.46 (1.95–3.26)	0.052
ESR, mm/h	10.00 (7.25–16.00)	12.00 (7.00–16.00)	9.60 (7.33–16.50)	0.628
CRP, mg/L	1.50 (0.41–3.55)	1.07 (0.33–3.28)	1.50 (0.67–4.54)	0.144
Therapy regimens, n (%)
Corticosteroid	119 (95.20)	60 (95.24)	59 (95.16)	0.984
Immunosuppressant	49 (39.20)	20 (31.75)	29 (46.77)	0.085
Intravenous immunoglobulin	16 (12.80)	9 (14.29)	7 (11.29)	0.616
Rehabilitation	4(3.20)	2 (3.17)	2 (3.23)	1.000
Spinal cord MRI, n (%)
LETM	83 (66.40)	36 (57.14)	47 (75.81)	0.027^*^
STM	42 (33.60)	27 (42.86)	15 (24.19)	
Initial EDSS, median (IQR)	5.00 (3.25–6.50)	4.50 (3.00–6.00)	5.50 (4.00–7.00)	0.025^*^
Relapse, *n* (%)	51 (40.80)	19 (30.16)	32 (51.61)	0.015^*^
Follow-up time, median (IQR)	37.87 (20.27–51.85)	39.13 (17.50–56.07)	37.45 (21.17–50.82)	0.680

*Continuous variables were presented as mean ± SD or median (IQR = 25th−75th percentile), and categorical variables were described as percentages (%)*.

*MHR, monocyte to high-density lipoprotein ratio; AQP4, aquaporin-4; TC, total cholesterol; TG, triglycerides; HDL, high density lipoprotein; LDL, low density lipoprotein; ESR, erythrocyte sedimentation rate; CRP, C-reactive protein; MRI, magnetic resonance imaging; LETM, longitudinally extensive transverse myelitis; STM, short-segment transverse myelitis; EDSS, Expanded Disability Status Scale*.

* *P < 0.05*.

### Correlations Between MHR and Disease Severity in Patients With NMOSD

**Figure 2:**
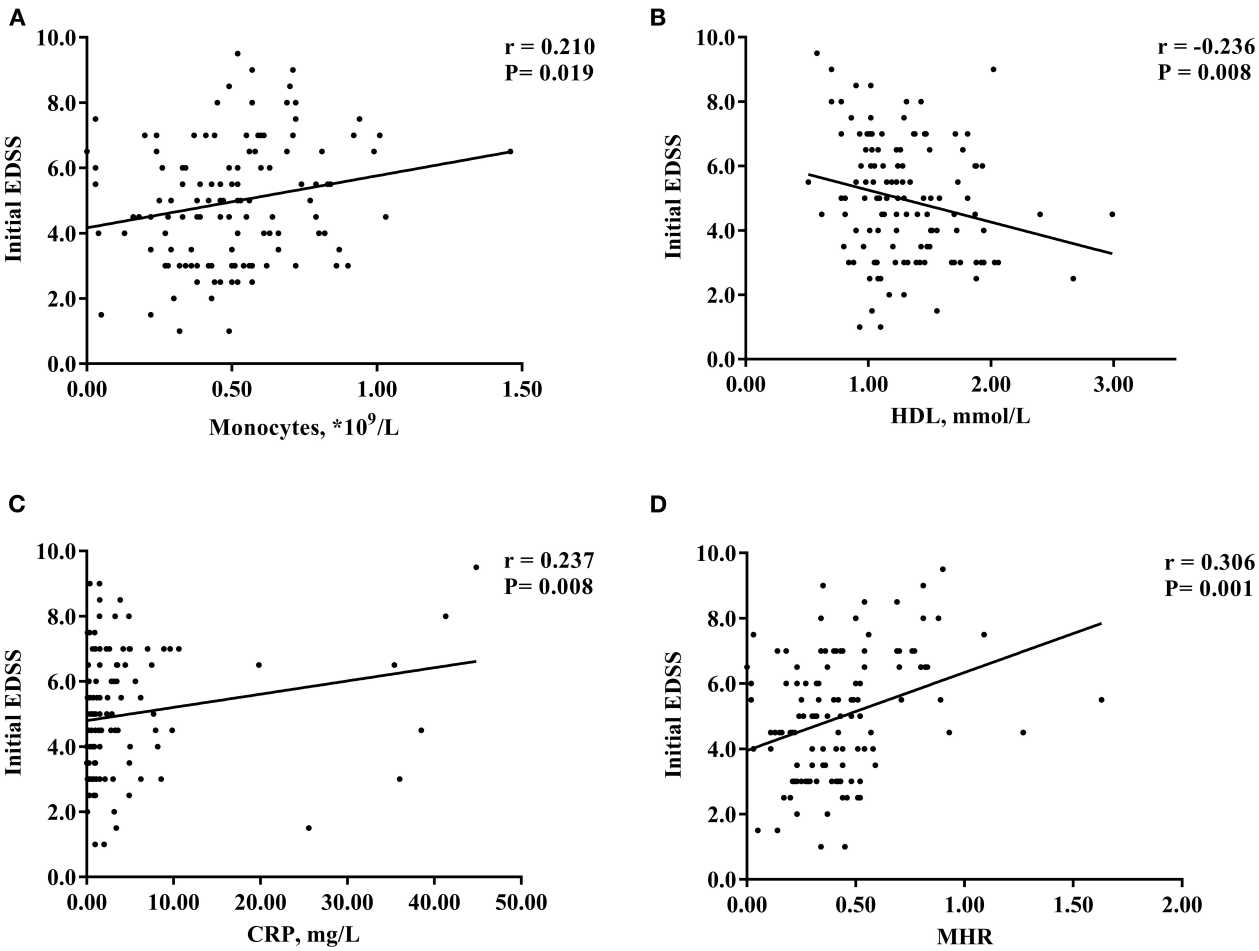
Scatter plot of the correlation between monocytes **(A)**, HDL **(B)**, CRP **(C)**, MHR **(D)** and initial EDSS scores. HDL, high-density lipoprotein; MHR, monocyte to high-density lipoprotein ratio; CRP, C-reactive protein; EDSS, Expanded Disability Status Scale. *P < 0.05.

Spearman correlation analysis showed that the levels of monocytes (*r* = 0.210, *P* = 0.019), HDL (*r* = −0.236, *P* = 0.008), and CRP (*r* = 0.237, *P* = 0.008) were significantly correlated with the initial EDSS score of patients with NMOSD, but the correlation was weak (Figures 2A–C). There was no obvious correlation between the blood lymphocyte count, ESR, blood lymphocyte to HDL ratio, and disease severity (*P* > 0.05) (Table 2). In addition, the MHR was positively correlated with the initial EDSS score (*r* = 0.306, *P* = 0.001) (Figure 2D).

**Table 2:** Correlation analysis of clinical parameters and initial EDSS scores.

	***r***	***P***
Monocytes, × 10^9^/L	0.210	0.019^*^
Lymphocytes, × 10^9^/L	−0.118	0.189
HDL, mmol/L	−0.236	0.008^*^
ESR, mm/h	0.022	0.806
CRP, mg/L	0.237	0.008^*^
Lymphocytes/HDL	0.014	0.874
MHR	0.306	0.001^*^

*EDSS, Expanded Disability Status Scale; HDL, high density lipoprotein; ESR, erythrocyte sedimentation rate; CRP, C-reactive protein; MHR, monocyte to high-density lipoprotein ratio*.

* *P < 0.05*.

Univariate logistic regression analysis showed that the MHR was significantly correlated with disease severity (OR = 9.55, 95% CI = 1.35–67.77, *P* = 0.024). HDL levels (OR = 0.49, 95% CI = 0.20–1.22, *P* = 0.126) and MRI of the spinal cord showing LETM (OR = 1.82, 95% CI = 0.82–4.03, *P* = 0.141) had a moderate influence on the initial EDSS score. However, age, gender, hypertension, anti-AQP4 antibody status, blood monocytes, lymphocyte count, TC, TG, LDL, ESR, CRP, and lymphocyte to HDL ratio were not significantly correlated with the initial EDSS score. In the multivariate model, MHR was an independent risk factor for disease severity (OR = 7.90, 95% CI = 1.08–57.82, *P* = 0.041) (Table 3).

**Table 3:** Binary logistic regression analysis of disease severity in patients with NMOSD.

	**Univariate analysis**		**Multivariable analysis**	
	**OR (95% CI)**	**P**	**OR (95% CI)**	***P***
Age at onset	1.02 (0.99–1.04)	0.201		
Gender, female	1.12 (0.39–3.20)	0.837		
Hypertension	1.66 (0.44–6.25)	0.456		
Anti-AQP4 status, positive	1.03 (0.47–2.26)	0.936		
Monocytes	2.72 (0.49–14.95)	0.251		
Lymphocytes	1.02 (0.70–1.49)	0.922		
TC	0.98 (0.74–1.31)	0.913		
TG	1.05 (0.68–1.64)	0.814		
HDL	0.49 (0.20–1.22)	0.126		
LDL	1.03 (0.70–1.52)	0.876		
ESR	0.99 (0.97–1.02)	0.600		
CRP	1.02 (0.97–1.09)	0.416	1.02 (0.96–1.08)	0.559
Lymphocytes/HDL	1.27 (0.78–2.08)	0.333		
Monocytes/HDL (MHR)	9.55 (1.35–67.77)	0.024^*^	7.90 (1.08–57.82)	0.041^*^
Spinal cord MRI, LETM	1.82 (0.82–4.03)	0.141	1.66 (0.74–3.77)	0.222

*OR, Odds ratio; CI, confidence interval; AQP4, aquaporin-4; TC, total cholesterol; TG, triglycerides; HDL, high density lipoprotein; LDL, low density lipoprotein; ESR, erythrocyte sedimentation rate; CRP, C-reactive protein; MRI, magnetic resonance imaging; LETM, longitudinally extensive transverse myelitis; MHR, monocyte to high-density lipoprotein ratio*.

* *P < 0.05*.

### Correlations Between MHR and Prognosis in Patients With NMOSD

**Figure 3:**
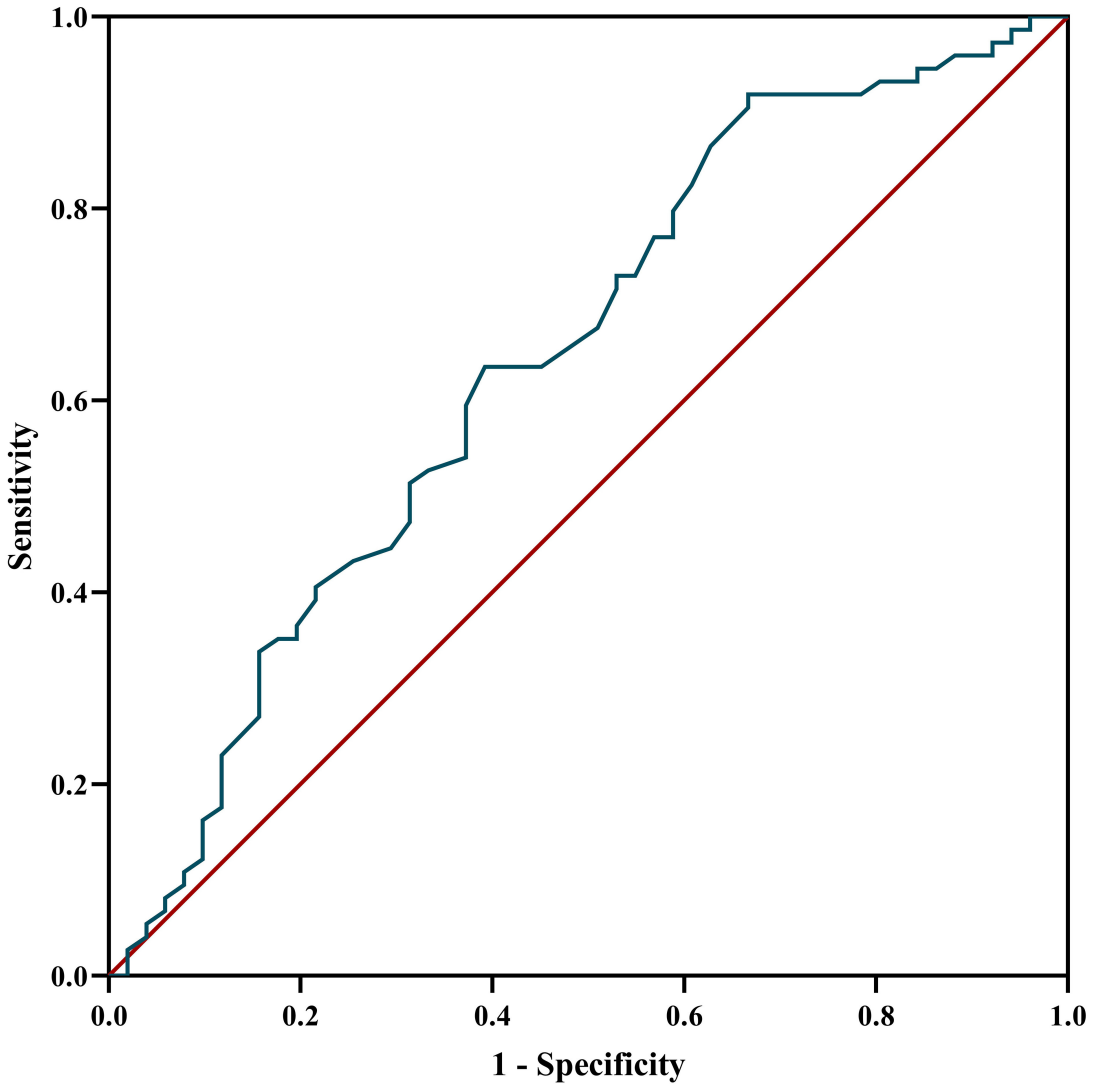
Kaplan-Meier curves categorized according to MHR. MHR, monocyte to high-density lipoprotein ratio. *P < 0.05.

Kaplan-Meier analysis was used to evaluate the correlation between MHR and the relapse rate of patients with NMOSD. MHR was a significant predictor of disease prognosis (log-rank P=0.046). The median relapse interval of the high MHR group was 24.40 months, whereas 50% of patients in the high MHR group relapsed 28.60 months after treatment (Figure 3).

**Figure 4:**
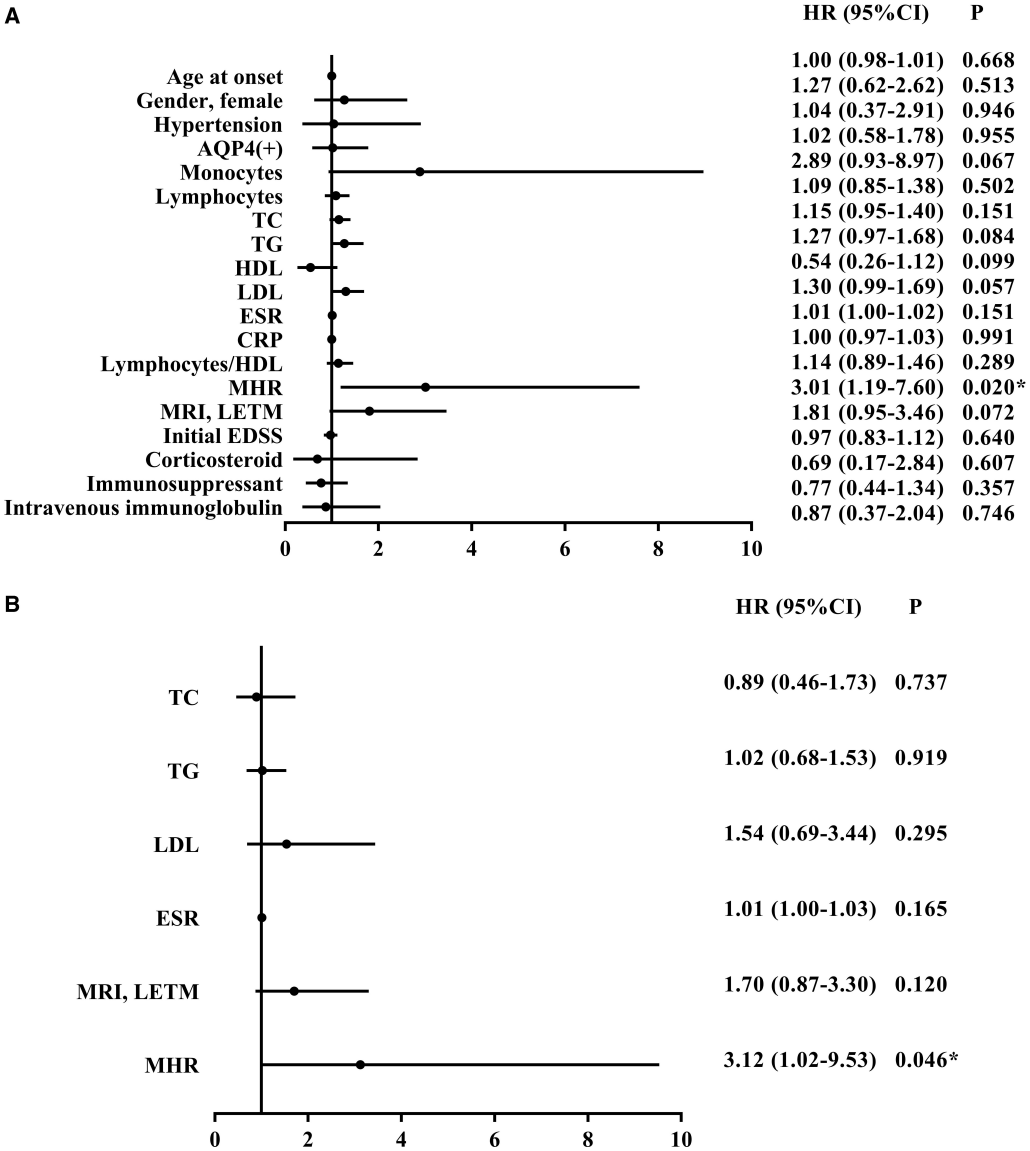
Univariate **(A)** and multivariate **(B)** cox survival analysis of potential factors associated with relapse of NMOSD patients. NMOSD, neuromyelitis optica spectrum disorders; AQP4, aquaporin-4; TC, total cholesterol; TG, triglycerides; HDL, high density lipoprotein; LDL, low density lipoprotein; ESR, erythrocyte sedimentation rate; CRP, C-reactive protein; MRI, magnetic resonance imaging; LETM, longitudinally extensive transverse myelitis; STM, short-segment transverse myelitis; EDSS, Expanded Disability Status Scale; MHR, monocyte to high-density lipoprotein ratio; HR, hazard ratio; CI, confidence interval. *P < 0.05.

Cox survival analysis was used to test the independent influence of MHRs on the relapse rate of patients with NMOSD. Univariate analysis showed that the MHR (HR = 3.01, 95% CI = 1.19–7.60, *p* = 0.020) was significantly correlated with relapse risk. However, there was no correlation between age, gender, hypertension, anti-AQP4 antibody, blood lymphocyte count, CRP, lymphocyte-to-HDL ratio, initial EDSS score, treatment plan, and relapse rate (*P* > 0.05). Blood mononuclear cell count, TC, TG, HDL, LDL, ESR, and MRI of the spinal cord showing LTEM had a moderate influence on disease relapse (Figure 4A). To eliminate these confounding factors, a multivariate Cox analysis model was established to obtain the corrected hazard ratio value. Because MHR is collinear with blood mononuclear cell count and HDL, these parameters were not included in the multivariate analysis. The results showed that the above confounding factors had no significant influence on the experimental results and that MHR was an independent risk factor for disease relapse (HR = 3.12, 95% CI = 1.02–9.53, *P* = 0.046) (Figure 4B).

**Figure 5:**
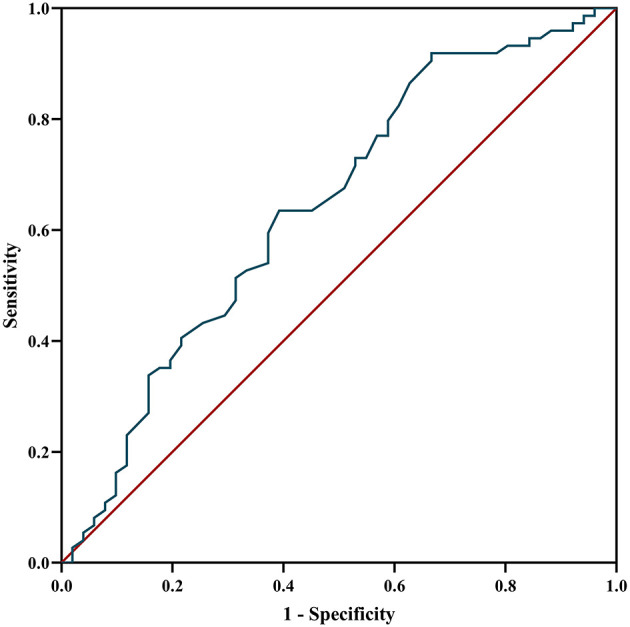
ROC curve analysis of the prediction of relapse based on MHR. ROC curve, receiver operating characteristic curve; MHR, monocyte to high-density lipoprotein ratio.

The ROC curve was used to analyze the predictive value of MHR for disease prognosis. The area under the curve was 0.642 (95% CI = 0.54–0.74, *P* = 0.007), the best cutoff value was MHR = 0.565, the sensitivity was 0.333, and the specificity was 0.919, showing a good predictive ability for disease relapse. When the MHR is higher than 0.565, the risk of relapse is high (Figure 5).

## Discussion

Previous studies have demonstrated the prognostic value of the MHR in immune-mediated diseases, and in this study, we evaluated the effect of the MHR on the disease severity and prognosis of patients with NMOSD. We found that high MHR was associated with more severe disease at onset and a higher relapse rate, and the MHR at the onset of NMOSD was positively correlated with the initial EDSS score. Further, MHR was found to be an independent predictor of the severity and prognosis of NMOSD, and the best cutoff value to predict prognosis was 0.565.

As important effector cells of the innate immune response, monocytes have many functions, including antigen presentation, phagocytosis, and cytokine production, and their role in autoimmune diseases has attracted increasing attention (10–13). The blood mononuclear cell count is closely related to the early clinical severity of MS and can be used as a prognostic index (11). In our study, the blood mononuclear cell count was positively correlated with the NMOSD initial EDSS score. Studies have shown that in NMOSD, anti-AQP4 antibodies can stimulate astrocytes to release chemokines (14) and enhance monocyte recruitment and activation. Activated monocytes promote the production of inflammatory cytokines, such as tumor necrosis factor α, interleukin (IL)-6, IL-1β, IL-12, and IL-23 (38), and increase the expression of costimulatory molecules, such as CD80, ICAM-1, and HLA-DR (39), whereas the level of anti-inflammatory cytokines (IL-10) decreases accordingly (40). Among these cytokines, IL-6 plays a key role in the pathogenesis of NMOSD, and treatment with IL-6 has shown clinical benefits in patients with NMOSD (41). Anti-AQP4 antibody is the main pathogenic antibody of NMOSD (42), but it alone is not enough to cause the disease. AQP4-specific T cells, especially Th17, can help peripheral blood B cells produce autoantibodies, induce tissue inflammation, and promote further injury to the central nervous system. IL-1β (43) induced by monocyte activation not only promotes Th17 differentiation but also causes blood–brain barrier leakage and enhances T cell migration (44); therefore, monocytes may play an important role in the pathological mechanism underlying NMOSD progression (45).

HDL is considered an anti-inflammatory factor that can prevent the production of pro-inflammatory cytokines and affect a series of immune cell reactions, including those involving macrophages and B and T lymphocytes (21). HDL is closely related to monocytes and can regulate the activation, adhesion, and migration of monocytes (46, 47). Apolipoprotein A-1, the main protein component of HDL, has a specific inhibitory effect on the production of inflammatory cytokines by monocytes through prevention of the activation of CD11b (48, 49). The production of anti-AQP4 antibodies in patients with acute NMOSD leads to the loss of astrocytes in specific areas of the central nervous system through complement-mediated cytotoxicity (50). It has been shown that astrocytes can produce apolipoprotein A-I (51) in rats; hence, the extensive loss of astrocytes leads to a significant decrease in the production of apolipoprotein A-I and a decrease in HDL levels. Previous studies have shown that the serum apolipoprotein A-1 levels in patients with NMOSD are significantly lower than those in healthy controls (22), and HDL levels are significantly lower during active disease than during remission. Further, dyslipidemia with low HDL is related to disease activity and disability in patients with AQP4 positive NMOSD (21). Similar results were observed in the present study. The lower the HDL levels, the higher the initial EDSS score of patients with NMOSD. Although TC, TG, and LDL had a moderate influence on disease prognosis in univariate analysis, no significant correlation was observed in multivariate analysis, and abnormal lipid metabolism in NMOSD has been confirmed by many studies. Some studies have found that compared with healthy controls, the levels of TC, TG, and LDL in patients with NMOSD are higher, and the level of TGs is positively correlated with poor recovery of first-time patients with NMOSD (20, 52). This, combined with our research results, suggests that early lipid-lowering treatment may play an important role in improving prognosis.

Compared with single monocytes and HDL levels, combining monocytes and HDL to form a new comprehensive inflammatory index that incorporates both the injury mechanism and protection mechanism, namely the MHR, may have greater clinical value as this biomarker can reflect the degree of inflammation and oxidative stress. Many studies have shown that MHR is closely related to Parkinson's disease (53), ischemic stroke (54), cardiovascular disease (23), metabolic syndrome (55), immune system disease (26), and rheumatic disease (27). Related studies have also reported that the neutrophil-to-lymphocyte ratio (NLR) was related to disease activity at the onset of MS (56). However, another study provided results that did not support the use of NLR as a marker of disease activity and disability in MS patients (57). The association between NLR and disease severity was also confirmed in NMOSD (58). However, the effect of MHR on the pathogenesis and prognosis of patients with NMOSD has not been previously reported. In our study, we found that MHR was positively correlated with the EDSS score of patients with NMOSD (*r* = 0.306, *P* = 0.001), and patients with high MHR had a higher relapse rate, which was an independent risk factor for disease severity and poor prognosis. Recently, it has been reported that the MHR of patients with multiple sclerosis with EDSS score ≥4 is significantly higher than that of patients with EDSS score <4, which proves that MHR is related to the severity and disability of MS and can be used as an independent indicator to predict disability (31). This previous report, combined with our research, suggests the potential role of MHRs in autoimmune demyelinating diseases of the central nervous system, and can also be used for risk assessment in the diagnosis and treatment of NMOSD. In our ROC curve analysis, MHR > 0.565 was associated with a high risk of relapse; therefore, intervention in the early stage of the disease process to reduce the MHR may have therapeutic potential, improve disease prognosis, and reduce the risk of relapse in clinical practice.

In our study, univariate analysis showed that spinal cord MRI showing LETM had a moderate influence on the initial EDSS score and prognosis of NMOSD, but there was no significant correlation in multivariate analysis, and previous studies have found that the length of spinal cord lesions is related to the initial severity of the disease and residual disability (59). The ability of MRI parameters to predict NMOSD prognosis requires further exploration. Consistent with previous studies, we found that the CRP was closely related to the disease, and the CRP level was positively correlated with the initial EDSS score (*r* = 0.237, *P* = 0.008) (60). The serum CRP level of patients with NMOSD was significantly higher than that of healthy individuals, which may be related to the oxidative stress and inflammatory reactions that occur during disease pathogenesis (61). CRP level is also related to the destruction of the blood–brain barrier and is a useful index for monitoring NMOSD disease activity (60, 61).

Our study also has several limitations worth noting. First, the retrospective study design has inherent defects. Second, this was a single-center cohort study with a relatively short follow-up time; therefore, a larger multi-center study with a longer follow-up period is warranted to confirm our results. Third, our hospital did not evaluate MOG antibody levels prior to 2019, and the lack of data prior to this date may have affected the research results. Fourth, selection bias should be considered. Because of incomplete test data, loss to follow-up, and other reasons, we only studied a small number of patients (125/650). Finally, some uncontrollable confounding factors may have affected the research results, such as smoking, drinking, eating habits, and drug intake.

In conclusion, our results showed that MHR was correlated with the severity of early NMOSD and could further predict disease relapse. This new biomarker, which is simple, reliable, economical, and easy to obtain, can also be used for NMOSD risk assessment in clinical practice and may facilitate earlier treatment and improvement of prognosis. Further studies are required to elucidate the specific mechanisms underlying the effect of MHR on NMOSD pathogenesis and disease progression.

## Data Availability Statement

The original contributions presented in the study are included in the article/Supplementary Material, further inquiries can be directed to the corresponding author.

## Ethics Statement

The studies involving human participants were reviewed and approved by the Ethics Committee of Zhengzhou University. Written informed consent to participate in this study was provided by the participants' legal guardian/next of kin.

## Author Contributions

JZ and YL contributed to conception and design of the research. YZho, CP, and YZha organized the database. JZ, YL, and HX performed the statistical analysis. JZ and KW conducted regular follow-up of all cases. JZ wrote the first draft of the manuscript. RD, ZG, and YJ undertook the task of revising the manuscript critically. All authors contributed to the article and approved the submitted version.

## Conflict of Interest

The authors declare that the research was conducted in the absence of any commercial or financial relationships that could be construed as a potential conflict of interest.

## Publisher's Note

All claims expressed in this article are solely those of the authors and do not necessarily represent those of their affiliated organizations, or those of the publisher, the editors and the reviewers. Any product that may be evaluated in this article, or claim that may be made by its manufacturer, is not guaranteed or endorsed by the publisher.
